# The impact of bariatric and metabolic surgery on the levels of circulating growth differentiation factor 15 in humans

**DOI:** 10.1007/s13105-026-01160-8

**Published:** 2026-03-31

**Authors:** Victor Yassuda, Beatriz Sequeira, Ana Luísa De Sousa-Coelho

**Affiliations:** 1 Algarve Biomedical Center Research Institute (ABC-Ri), Universidade do Algarve, Faro, 8005-139 Portugal; 2 Faculdade de Medicina e Ciências Biomédicas (FMCB), Universidade do Algarve, Faro, 8005-139 Portugal; 3 Escola Superior de Saúde, Universidade do Algarve (ESSUAlg), Faro, 8005-139 Portugal

**Keywords:** Obesity, Growth Differentiation Factor 15, Hepatokines, Obesity, Bariatric and metabolic surgery

## Abstract

Obesity is a known risk factor cardiovascular disease, type 2 diabetes, metabolic dysfunction-associated steatotic liver disease (MASLD) and steatohepatitis (MASH), characterized by a state of chronic inflammation. In this stress context, many hepatokines and adipokines may be released, including Growth Differentiation Factor 15 (GDF15). GDF15 is involved in the regulation of appetite and metabolism, showing potential for the treatment of obesity and related comorbidities. This review aimed to evaluate the impact of bariatric and metabolic surgery (BMS) in the levels of circulating GDF15 in individuals with obesity. Considering the studies available, reporting GDF15 levels before and 12 months after BMS, the profile of GDF15 changes was not a consensus, and was not fully explained by differences in the characterization of the population in each study. This review also discusses the multiple sources of GDF15 in obesity and in response to its surgical treatment, where pathological, pharmacological, and behavioral factors may all be important contributors.

## Key points

• Growth Differentiation Factor 15 (GDF15) is a potential biomarker and therapeutic target in metabolic diseases.

• GDF15 is a cytokine secreted by several tissues in response to multiple stress conditions.

• GDF15 may be involved in the outcomes of bariatric and metabolic surgery (BMS) for the treatment of obesity.

## Introduction

Obesity is defined by the excess of adipose tissue and increased body mass index (BMI). Rather than relying solely on BMI, clinical obesity is diagnosed when excess fat negatively impacts the body’s organs and tissues [1]. The accumulation of fat in the adipocyte is associated with the production of reactive oxygen species, oxidative stress, and the establishment of state of chronic inflammation [2]. Increased visceral adiposity is a key-point of the pathogenesis of many comorbidities, such as type 2 diabetes (T2D), cardiovascular diseases and metabolic dysfuction-associated steatotic liver disease (MASLD), characteristics of the metabolic syndrome (MetS) [3]. In this inflammatory context, various stress-related cytokines are released, mainly from hepatic and adipose tissues, contributing to a complex network of metabolic and inflammatory processes [4, 5]. Despite the recent pharmacological perspectives, bariatric and metabolic surgery (BMS), namely Roux-en-Y Gastric Bypass (RYGB) and sleeve gastrectomy (SG), is still one of the most effective strategies for the obesity treatment, accounting improvements in many obesity-related diseases [6–8].

Growth differentiation factor 15 (GDF15), also known as macrophage inhibitory cytokine (MIC-1), is a member of the glial cell line-derived neurotrophic factor (GDNF) family, within the transforming growth factor beta (TGF-β) superfamily [9]. GDF15 is a stress-induced cytokine that is produced and secreted by multiple tissues, namely striated muscles, liver and adipose tissue [10–13]. Its major role is related to the regulation of appetite and metabolism [5, 14]. After being released, GDF15 crosses the blood-brain barrier and binds to the GDNF α-like receptor (GFRAL) and the co-receptor REarranged during Transfection (RET), limited in the area postrema (AP) and nucleus of the solitary tract (NTS), forming the complex GDF15-GFRAL [15–17]. Recent evidence showed that increased levels of circulating GDF15, contributes to the reduction of fat mass and hepatic steatosis in diet-induced obesity mouse models [18], to the increase in the adipose tissue lipolysis in humans [19], and to maintaining energy expenditure in skeletal muscle during caloric restriction [20], extending its impact beyond its anorexic affect, and emphasizing its high relevance in the obesity field. Additional studies showed that inducing endogenous GDF15 may serves as a protective mechanism against metabolic dysfuction-associated steatohepatitis (MASH) progression, highlighting the potential of GDF15 as a therapeutic target for both MASH-related metabolic deterioration and liver fibrosis [21, 22].

GDF15 analogs have garnered significant attention due to their pharmacological potential in obesity therapy [23]. These compounds exhibit a distinct mechanism of action compared to glucagon-like peptide 1 (GLP-1) analogs, which are currently widely used in clinical settings for both diabetes and obesity treatment, demonstrating an additive potential when combined [24]. In addition, recent preclinical studies have shown promising results in the development of long-acting dual agonists (GLP-1/GDF15 receptors) showing significant reductions in body weight (BW) in mice [25]. To contribute to the understanding of GDF15 as a molecule with high potential for anti-obesity treatments, the main goal of this review is to evaluate the impact of BMS in the levels of circulating GDF15 and to interpret its potential involvement in the sustained weight loss and metabolic improvements achieved after BMS in humans living with obesity.

## Materials and methods

For the search of suitable articles for this review, a combination of keywords was used (Table 1). This was performed at Web of Science, Scopus, and PubMed, carried out from June 14th to June 30th, 2023. An additional and final confirmatory search was performed before the final preparation of the manuscript in June 2024.

**Table 1:** Search strategy

Molecule		Intervention^1^
GDF-15		Bariatric surgery
		Metabolic surgery
GDF15	AND	Obesity surgery
		Gastric bypass
Growth differentiation factor 15		RYGB
		Sleeve gastrectomy

^1^ Search performed only in Title/Abstract for each database

Inclusion and exclusion criteria were established for the selection of articles (Table 2). Articles were considered from inception, and neither geographic location nor the sample size were used as exclusion criteria.

**Table 2:** Inclusion and exclusion criteria

Inclusion	Exclusion
Individuals with obesity who performed any kind of bariatric surgery (BMS)	Studies that included structured physical activity, without a non-exercised control
Circulating levels of GDF15 measured before BMS and in, at least, one defined moment (follow-up, FU) after BMS	Measurement of GDF15 levels only in tissues
Original articles	Cross-sectional studies, clinical cases, clinical trials without results
Articles in English, Portuguese, Spanish	Full text unavailability

Rayyan platform was used to eliminate all duplicates from the three databases [26]. By applying the inclusion and exclusion criteria, the abstracts and titles were initially screened. Upon screening all the articles, those that did not show a primary reason for exclusion, were selected to full text reading. This process was performed by two researchers independently and confirmed by a third researcher. After the final decision for inclusion, data regarding title, first authors surname, journal, year of publication, characteristics of the study population, type of surgery, time of follow-ups, and the levels of circulating GDF15 at different timepoints, were collected from each article and organized in tables. When not stated in the original text, values were obtained directly from the published plots, as indicated in figure legends.

## Results

### Search results and study selection

**Fig. 1:**
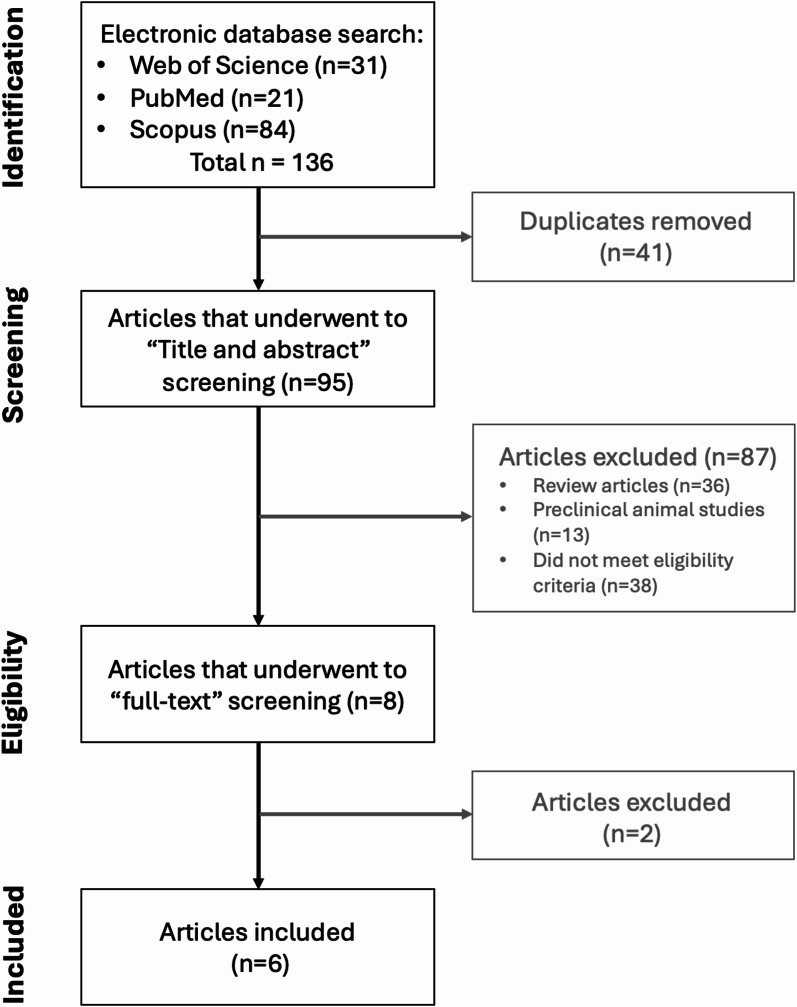
Flow diagram of the search. On the identification phase, from the three databases used to search the articles, the number (n) of the results and the final summation was 136. After removing the duplicates, it resulted in 95 articles. On the screening phase, all review articles, animal studies and articles irrelevant to the main subject were excluded. From the 8 remaining articles for full text reading 2 articles were excluded because the results described did not fulfil the inclusion criteria. After the process of exclusion, 6 articles were considered

Following the search strategy, a total of 136 results were initially obtained (Fig. 1). After removing the duplicates, this number was reduced to 95 articles. Hereupon, based on the title and abstract, 36 review articles, 13 articles with animal studies, and 38 unrelated to the subject, were excluded. For the remaining 8 articles, the full text was analyzed.

After full text reading, 2 articles were additionally excluded based on the fact the results obtained would not be comparable with the other selected studies. In one case, patients submitted to BMS were all subjected to exercise training programs after BMS, meaning there was not a group of patients without the exercise intervention [27]. In the other case, GDF-15 levels were evaluated in patients at an undefined follow-up time, ranging from 6 to 12 months postoperatively [28]. Finally, from the initial search, 6 articles were selected for detailed analysis (Fig. 1).

The 6 articles included for anlysis were published between 2011 and 2023 (Table 3), although mostly in the last 6 years (5 of 6 studies were published from 2019 to 2023), suggesting a novel interest in the topic. The articles will be referred hereafter by the surname of the first author and the year of publication, namely Chaiyasoot 2023 [29], Schmid 2022 [30], Salman 2021 [31], Dolo 2020 [32], Kleinert 2019 [33] and Vila 2011 [34].

**Table 3:** General information of the selected articles, referring title, first author, location of the study, journal and year of publication

Title	First author	Location	Year	Journal	Ref.
Alteration of BDNF, SPARC, FGF-21, and GDF-15 circulating levels after 1 year of anti-obesity treatments and their association with 1-year weight loss	K Chaiyasoot	Bangkok, Thailand	2023	Endocrine	[29]
Circulating Adipokines and Hepatokines Serve as Diagnostic Markers during Obesity Therapy	A Schmid	Giessen, Germany	2022	International Journal of Molecular Sciences	[30]
Changes in Plasma Growth Differentiation Factor-15 After Laparoscopic Sleeve Gastrectomy in Morbidly Obese Patients: A Prospective Study	A Salman	Cairo, Egypt	2021	Journal of Inflammation Research	[31]
Effect of sleeve gastrectomy on plasma growth differentiation factor 15 (GDF15) in human.	PR Dolo	Jiangsu, China	2020	The American Journal of Surgery	[32]
Effect of bariatric surgery on plasma GDF15 in humans	M Kleinert	Copenhagen, Denmark	2019	Am J Physiol Endocrinol Metab	[33]
The Relationship between Insulin Resistance and the Cardiovascular Biomarker Growth Differentiation Factor-15 in Obese Patients	G Vila	Vienna, Austria	2011	Clinical Chemistry	[34]

## Characterization of the population in study

Different variables were extracted from each article, namely the baseline characteristics of the population of each study (Table 4). The size of the population in study was variable, ranging from cohorts of 21 to 81 patients, with age averaging between 33 and 45 years old. Overall, most participants were female, although Dolo 2020 [32] and Salman 2021 [31] included 62% and 50% of male patients, respectively. Various comorbidities were identified, namely T2D, which was present, although at different proportions, in all populations of the articles selected. Hypertension and dyslipidaemia were other of the comorbidities diagnosed in these individuals, also with variable proportions in each case. At each study, all participants were submitted to one of two main types of BMS, either sleeve gastrectomy (SG) [31, 32] or Roux-en-Y gastric bypass (RYGB) [29, 30, 33, 34] (Table 4).

**Table 4:** Description of the main baseline characteristics of the participants of each study

ID [Ref.]	Sample size (*n*)	Mean age (years old)	Sex distribution	Comorbidities	Type of BMS
Chaiyasoot 2023 [29]	41	[39]^#^	[63% F]^#^	[T2D (26.3%), HTN, DLP, OSA]^#^	RYGB
Schmid 2022 [30]	79	41	82% F	T2D (24%), HTN (65%), DLP (41%), CVD (4%)^&^	RYGB
Salman 2021 [31]	68	42	50% F	T2D (28%), HTN (40%), DLP (22%), OSA (16%)	SG
Dolo 2020 [32]	21	33	38% F	T2D (29%)	SG
Kleinert 2019 [33]	47	46	64% F	T2D (47%)	RYGB
Vila 2011 [34]	28	43	89% F	[T2D (12%)]^#^	RYGB

^#^Values available from the entire cohort, not specifically for the group of patients submitted to BMS. ^&^Values obtained from a previous publication with the same population [35]. The percentages of patients with comorbidities are shown only when available (values were rounded to the unit). Abbreviations: F, female; T2D, type 2 diabetes; HTN, hypertension; DLP, dyslipidemia; OSA, obstructive sleep apnea; CVD, cardiovascular disease; BMS, bariatric and metabolic surgery; RYGB, Roux-en-Y gastric bypass; SG, sleeve gastrectomy

Before surgery, the BW and BMI of the individuals averaged between 124.6 and 149.4 Kg, and 41.4 and 50.2 Kg/m^2^, respectively (Table 5). Only 1 study evaluated body fat percentage, namely the work of Schmid and colleagues. As anticipated, 1 year after BMS, a decrease in body fat was observed (from 52 to 35.5%) [30]. We noticed some studies rather performed correlations between the weight loss clinical outcomes and changes in GDF15 levels, not presenting the data for the main clinical outcomes after BMS independently of the intended analysis.

**Table 5:** Changes in body weight and body mass index, and main clinical outcomes reported

ID [Ref.]	BW (Kg)		BMI^1^ (kg/m^2^)		Main clinical outcomes
	Baseline	After 12 months	Baseline	After 12 months	After 12 months
Chaiyasoot 2023 [29]	134 ± 31	102 ± 26	50.2 ± 9.3	38.0 ± 7.4	↓ TG, TC; ↑ HDL-c; ↓ FPG, HbA1c, fasting insulin, HOMA-IR
Schmid 2022 [30]	149.4	94.6	51.7	33.1	↓ NAFLD fibrosis score
Salman 2021 [31]	136.67 ± 11.6	98.52 ± 8.8	44.8 ± 3.5	32.37 ± 3.6	Reversed 14.7% T2D, 25% HTN, 16.2% DLP
Dolo 2020 [32]	127.7 ± 31.3	-	43.5 ± 7.1	-	-
Kleinert 2019 [33]	124.6 ± 2.85^&^	-	41.4 ± 0.7^&^	-	-
Vila 2011 [34]	128.3 ± 3	95 ± 3	[47.1 ± 0.6]^#^	-	↓ fasting insulin, HOMA-IR, TC, LDL-c, CRP

^&^Values calculated as weighted average with the available data from the groups. ^#^Values available from the entire cohort, not specifically for the group of patients submitted to BMS. ^1^average BW and BMI ± SD or SE (standard deviation or error mean) is shown, except for Schmid 2022, for which a measure of variability or dispersion was not shown [30]. Abbreviations: BW, Body Weight, BMI, Body Mass Index; TG, tryglicerides; TC, total cholesterol; HDL-c, High Density Lipoproteins-cholesterol; FPG, fasting plasma glucose; HbA1c, glycated heamoglobin; HOMA-IR, Homeostatic Model Assessment of Insulin Resistance; NAFLD, non-alcholoic fatty liver disease; T2D, type 2 diabetes; HTN, hypertension; DLP, dyslipidemia; LDL-c, Low Density Lipoproteins-cholesterol; CRP, C-reactive protein

## Evaluation of the circulating levels of GDF15 before and after BMS

For the evaluation of the circulating levels of GDF15, specific assays were performed to analyze the samples collected, mainly an enzyme-linked immunosorbent assay (ELISA) (Table 6). Authors used either serum or plasma. Most studies reported that fasting blood was collected. While the feeding state of the patients before collection was not disclosed in 2 manuscripts [30, 34], based on a previous study with the same sample [35] and the fact that most biochemical parameters were shown in the fasting state [34], it is highly likely that all measurements were performed in samples from fasted patients (Table 6).

**Table 6:** GDF15 assays and type of sample used

ID [Ref.]	Samples/GDF15 Assays		
	State	Type	Assay (brand)
Chaiyasoot 2023 [29]	Fasting	Plasma	Bead-based multiplex assay (Human Myokine Panel, Merck)
Schmid 2022 [30]	-	Serum	ELISA (R&D systems)
Salman 2021 [31]	Fasting	Serum	ELISA (Cloud-Clone Corp)
Dolo 2020 [32]	Fasting	Plasma	ELISA (Cloud-Clone Corp)
Kleinert 2019 [33]	Fasting	Plasma	ELISA (R&D Systems)
Vila 2011 [34]	-	Plasma	ELISA (R&D Systems)

Abbreviations: ELISA, enzyme-linked immunosorbent assay

In all selected studies, the levels of circulating GDF15 were evaluated before and after BMS at different timepoints. The average levels of circulating GDF15 at baseline (before BMS) were very variable, between 215.1 and 1165 pg/mL, although most mean values are found between 400 and 500 pg/mL (Table 7).

**Table 7:** Mean levels of circulating GDF15 (pg/mL) before, and after BMS at different timepoints

**ID** [Ref.]	Baseline	After 1 week	After 1 month	After 3 months	After 12 months	After 2.5–4.5 years
Chaiyasoot 2023 [29]	1165 (674;1864)	-	-	-	606 (444;1381)	-
Schmid 2022 [30]	481 ± 416	-	-	-	319 ± 200	-
Salman 2021 [31]	409.93 ±119^1^	-	-	-	699.941 ±193.5^1^	-
Dolo 2020 [32]	215.1 ± 199.9^1^	-	301.9 ± 135.2^1^	338.86 ± 131.14^1^	329.39 ± 152.1^1^	-
Kleinert 2019 [33]	487 ± 28^2^	689 ± 45^2^	-	554 ± 37^2^	566 ± 37^2^	630± 50^2^
Vila 2011 [34]	474 ± 31^2^	-	-	-	637 ± 52^2^	-

If neither SD or SE were available, the interval of values (minimum; maximum) is shown. ^1^average ± SD; ^2^average ± SE. Abbreviations: GDF15, growth differentiation factor 15; BMS, bariatric and metabolic surgery; SE, standard error; SD, standard deviation

**Fig. 2:**
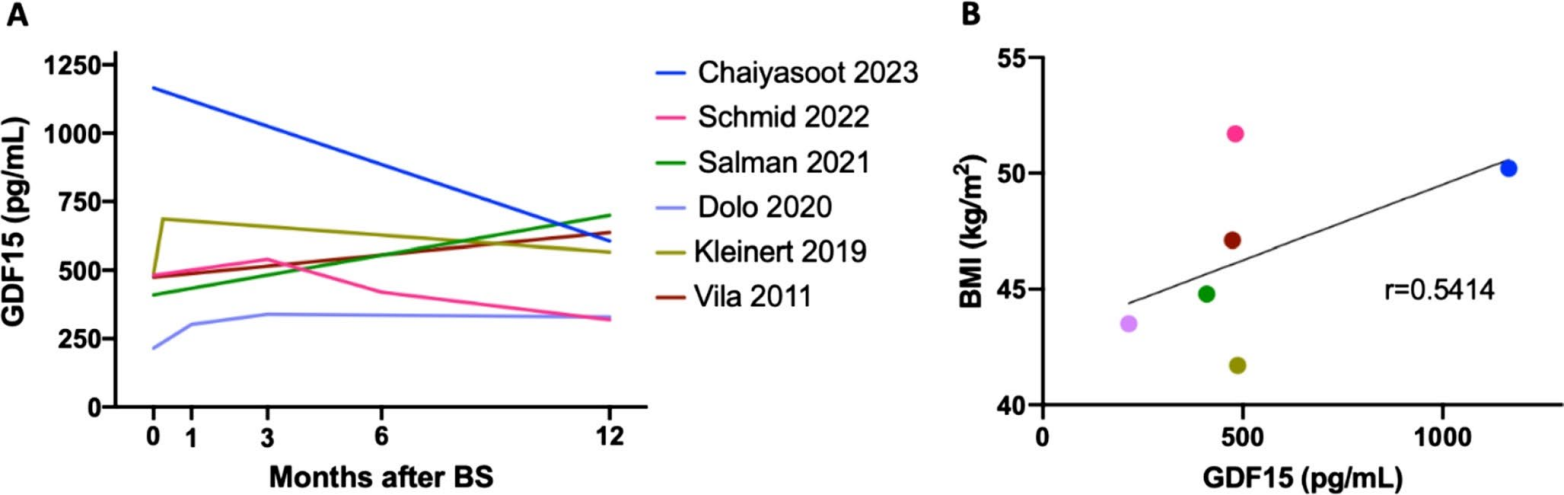
Post-BMS dynamics of GDF15 and baseline correlation with BMI. **A**. Mean levels of circulating GDF15 (pg/mL) before (0), and 1week (0,25), 1 month, 3 months and 12 months after BMS. The values plotted for the 3 and 6 months follow-up from Schmid 2022 are an approximation obtained from the graphic in the respective article [30]. **B**. A Pearson correlation was performed between the mean GDF15 levels and the mean BMI values at baseline in each study, revealing a moderate association (r = 0.5414). Abbreviations: GDF15, growth differentiation factor 15; BMS, bariatric and metabolic surgery; BMI, body mass index. Abbreviations: GDF15, growth differentiation factor 15; BMS, bariatric and metabolic surgery; BMI, body mass index

When comparing the levels from baseline to the longest follow-up (1 or 2.5 to 4 years), 4 studies showed an increase while the other 2 showed decreased levels of GDF15 compared to baseline (Table 7). The earliest measurement was at 1 week after BMS, showing an increase of 41% compared to baseline [33]. From this same study (Kleinert 2019), at 2.5 years after BMS, a value of 630 ± 50 pg/mL was obtained, reflecting an early increase followed by a relatively stable plateau overtime. Since all studies measured GDF15 1 year after BMS, a plot with all values until that timepoint was prepared, including the values from the 3 and 6 months follow-up from Schmid 2022 [30], which were only available from their original plots (Fig. 2 A).

The results from Chaiyasoot 2023 showed not only the highest levels of GDF-15 at baseline (more than doubling the levels of all other articles) [29], as also the highest drop from baseline to the 1 year follow-up, achieving almost a 50% decrease (Table 7; Fig. 2A). When combining the results of all studies, the BMI *vs*. GDF15 at baseline only partially correlated (Fig. 2B), reflecting some independence of the levels of GDF15 and the stage of obesity before the surgical intervention.

## Correlation of GDF15 with other parameters

In most studies, the authors aimed to find correlations or associations between the changes in GDF15 levels with the characteristics of the patients and their changes overtime, namely the BMI and BW loss (Table 8).

**Table 8:** Statistically significant correlations between changes in the GDF15 levels and weight or BMI loss after BMS

ID [Ref.]	Pearson (time after BMS)	*p* value
Salman 2021 [31]	*r* = 0.535 (12 months)^1^	*< 0.001*
Dolo 2020 [32]	r^2^ = 0.204 (3 months)^2^	*< 0.05*
Kleinert 2019 [33]	r^2^ = 0.211 (2.5–4.5 years)^1^	*0.0055*
Vila 2011 [34]	*r* = 0.541 (12 months)^2^	*0.004*

^1^weight loss; ^2^BMI loss. Abbreviations: Abbreviations: GDF15, growth differentiation factor 15; BMS, bariatric and metabolic surgery; BMI, body mass index

Salman 2021 found that the magnitude of the increase in the levels of GDF15 was correlated with the BW loss at 1 year after BMS [31]. In the study by Dolo 2020, exclusively at 3 months after BMS (but not at any other timepoint), increased levels of GDF15 were correlated with the degree of the BMI loss [32]. Kleinert 2019 also found that the changes in GDF15 correlated with the grade of BW loss, after 2.5 to 4 years after BMS [33]. In agreement, Vila 2011 showed that the increase in GDF15 1 year after BMS was associated with the observed decreased in the BMI [34].

While in the previous referred articles (Table 8), GDF15 levels were increased, in the 2 most recent articles the levels of GDF15 were on average decreased after BMS [29, 30]. Not specifically shown for the surgical treatment of obesity, the study by Chaiyasoot 2023 [29], found that for their entire cohort of patients with obesity subjected to either lifestyle education intervention (LEI) alone or LEI plus pharmacotherapy (topiramate, liraglutide or orlistat) or LEI plus BMS, the decrease in GDF15 (Fig. 2 A) was associated with a greater percentage of weight loss at 12 months after the obesity treatment. Schmid 2022 also showed results applied to the whole study cohort (both groups of low-calorie diets (LCD) and of patients submitted to BMS) [30]. In this case, they found that GDF15 was not able to discriminate patients with a higher or a lower body fat loss after the anti-obesity program or surgical intervention. Interestingly, although in average the levels of GDF15 decreased (Fig. 2 A), they found that the patients showing increased levels of GDF15 at the 12 months follow-up (compared to baseline), showed higher levels of the Fibrosis-4 index for liver fibrosis (FIB-4 score), compared to those patients that showed a reduction in the levels of GDF15 post intervention. From these observations, the authors highlighted that GDF15 could be an adequate marker to differentiate patients with advanced hepatic fibrosis from those without [30]. Analyzed specifically in the BMS group, Schmid 2022 also showed that changes in GDF15 from baseline to post-surgery positively correlated with HbA1c changes. Consequently, GDF15 was proposed as an adequate T2D classifier, exclusively in patients submitted to BMS, but not to the LCD program [30].

## Discussion

GDF15 is a cytokine released from various types of tissues physiologically and under different kinds of stress, where increased levels have been associated with disease states. As a result of the weight loss induced by the bariatric and metabolic surgery (BMS), many tissues undergo substantial changes, not only the adipose tissues but also the muscle and liver, common sites of GDF15 production. Although the changes in GDF15 expression might be a consequence of those tissue variations, its levels in circulation might contribute to the sustained weight loss after BMS, by increasing lipolysis and modulation of the appetite, reducing the individuals’ food intake [14]. Following a systematized search, we found not many studies in humans that evaluated GDF15 circulating levels pre and post BMS. From the six longitudinal studies available and included in this review, the directionality of the changes in circulating GDF15 was not a consensus (Fig. 2 A). Adding to the complexity of our findings, while the current manuscript was being reviewed, Urones and colleagues showed that the levels of GDF15 significantly decreased from baseline to 12 months after BMS, in both male and female individuals [36]. Globally, circulant GDF15 decreased from 904.09 ± 65.23 to 653.71 ± 39.57 pg/mL, which would agree with the results from Chaiyasoot [29], which showed the highest values at baseline (when compared to all other studies) and about a one-third decrease in GDF15 values. Nevertheless, another study to be mentioned, published out of our extended search period, showed that the levels of GDF15 in circulation increased after BMS, specifically in patients without metabolic syndrome, while in those with metabolic syndrome GDF15 remained unchanged from baseline (patients with obesity) to 12–16 months after SG [37]. These results highlight the relevance and need of an individualized and paired analysis, along and exhaustive characterization of the patients, namely their main obesity-associated diseases. However, such additional recent publications add to the conflicting data currently available.

GDF15 elevated levels were previously described in diabetes, inflammatory states, and cardiovascular diseases [14]. These are conditions that often overlap with obesity. The serum concentration of GDF15 is elevated in both humans and animals with obesity, and is generally considered a consequence of the condition rather than a contributing factor [38]. In a recent review, Breit et al. proposed GDF15 ranges associated with different pathologies [39]. While considering normal values approximately between 200 and 1200 pg/mL, values associated with obesity, diabetes, metformin intake, cardiovascular disease, anorexia and early stages of cancer, varied between 800 and 3000 pg/mL. As anticipated, all the patients from the studies included in this review had criteria for BMS, i.e., were living with obesity. Nevertheless, only the baseline values of Chaiyassot 2023 cohort showed values within the disease range (over 800 pg/mL), although in the overlapping range with those considered normal (below 1200 pg/mL), with a mean value of 1165 pg/mL [29]. This explanation is highly unlikely, but it is worth mentioning that the high baseline levels observed in Chaiyassot 2023, coincide with the only study that used a different type of assay for the analysis of GDF15 (Table 6). Intricately, the populations with either the highest or the lowest mean BMI (Schmid 2022 and Kleinert 2019, respectively [30, 33]), showed similar baseline levels of GDF15 (around 500 pg/mL), performing as putative outliers in the correlation analysis performed (Fig. 2B). Different baseline levels might contribute to different changes after BMS. Although age may be a predictor of high baseline GDF15 [40], in all studies patients had similar age. GDF15 was previously proposed as a biomarker of biological age [41], and, very recently, plasma proteomics found GDF15 among the eight proteins which positively associated with brain aging [42]. While positively associated with higher risk of brain disorders, it was negatively associated with normal walking pace [42].

When interpreting GDF15 levels in individuals with obesity, the presence of comorbidities must be considered. In the above cited review [39], GDF15 ranges associated with liver dysfunction were not proposed. Patients with obesity often show liver abnormalities, namely accumulation of fat in the liver, which induces local inflammation and fatty liver disease. MASLD can progress from simple steatosis to MASH, fibrosis, cirrhosis, and ultimately, to hepatocellular carcinoma (HCC) [43]. The stage and progression of such hepatic dysfunction positively correlates with GDF15 levels, where hepatocytes could be the main source of GDF15 expression and secretion, independent of obesity and adiposity [38, 44]. It was recently reviewed that after BMS, most patients ameliorated their liver function and showed improved biochemical parameters, such as a decrease in the circulating levels of aspartate aminotransferase (AST) and alanine aminotransferase (ALT) [8, 45, 46]. With all this evidence, it would be expected that GDF15 levels would consistently decrease after BMS, which was observed only in 2 of the studies [29, 30]. Among the articles included in this review, only 2 evaluated hepatic aminotransferases before and after BMS, both of which found a significant relationship between the decrease in hepatic parameters after surgery and GDF15. However, while it was associated with a decrease in GDF15 in Schmid 2022 [30], it was with an increase in GDF15 in Salman 2021 [31]. Moreover, since when disclosing the patients’ comorbidities, the information regarding liver function was lacking in most studies, it hampers a deeper discussion about being the liver an important contributor of the changes observed. While showing the prevalence of patients with T2D (Table 4), only 3 studies reported the inclusion of patients with HTN (and only 2 of them referred the % of patients). Similarly, only 2 studies reported the inclusion of patients with dyslipidemia (and only 2 of them referred the % of patients). Altogether, this limits the overall understanding of the influence of comorbidities in the levels of circulating GDF15 in patients with obesity.

Also relevant to be noted, is not only the need of a detailed characterization of the obesity related disorders, but also the pharmacological treatments adopted for each patient, which is crucial to well analyze the impact of BMS in the circulating levels of GDF15. In all articles, patients with T2D were identified at baseline, though in different proportion in each cohort. Although the medication was not fully disclosed in the studies, the drug most adopted for T2D treatment is metformin [47], which may also be used in additional prevalent diseases associated with obesity, such as prediabetes and polycystic ovary syndrome (PCOS) [48, 49]. It was recently described that metformin administration induces an increase in GDF15 levels, being the intestinal epithelium its main source [50–52]. Since patients may experience full remission of T2D after BMS [53, 54], it means many patients can maintain their glycemic control without the use of antidiabetic drugs. However, the rate of T2D remission or metformin withdrawal in the populations of each study is not clear, limiting the possibility of better exploring these points.

Increased muscle transcriptional and serum levels of GDF15 were observed after acute exercise [12, 55], which could be related to exercised-induced inflammation. This means an additional relevant source of GDF15 in these patients is the skeletal muscle. Indeed, according to protocol [6], there is a significant increase in physical exercise by patients who have undergone surgery. However, this has not been assessed in any study, neither at baseline nor post-op. Previous research showed somehow dissimilar results, where individuals subjected to the same exercise protocols, either showed increased or decreased GDF15 levels [56, 57]. Others even showed an inverse correlation between an active lifestyle and GDF15 circulating levels [58]. In any case, it is suggested that skeletal muscle might not be the main source of GDF15 after exercise, but rather the liver [12, 59]. On the other hand, patients after BMS may lose significant amount of skeletal muscle mass [60], which may also affect the expression levels and secretion of GDF15 as a myokine, although patients’ body composition distribution was not available in these studies. Of note, elevated levels of GDF15 were previously positively associated with low muscle mass and sarcopenia [61, 62].

Finally, one of the notable sources of GDF15 in obesity is the adipose tissue, released by macrophages that infiltrate and accumulate in this tissue, which contribute to the state of systemic low-grade inflammation [63]. In all cases, as anticipated, patients lost extensive amounts of body mass after BMS, which is anticipated to be mainly due to losing adipose tissue [6], as indicated by Schmid [30]. The disparity in the directionality of circulating GDF15 levels after BMS suggests, however, that the adipose tissue may not be its main source.

GDF15 transcritption may be regulated by different transcription factors and signalling pathways (endoplasmic reticulum (ER) stress, the unfolded protein response (UPR), and AMP-activated protein kinase (AMPK) signaling), related with stress and inflammatory processes, along metabolic derangements [38, 64]. There is also some evidence that an acute elevation of free fatty acids (FFA) can regulate GDF15 expression and secretion levels [65, 66]. Despite a transient increase in plasma FFA may occur in the short-term after BMS due to lipolysis and fat mobilization, their circulating levels will generally decrease and normalize after body weight stabilization, depending on the time of follow-up considered [67, 68]. Since FFA were not evaluated in the selected studies (Table 3), their role or contribution in the regulation of GDF15 levels after BMS in the long-term can’t be currently further explored. As part of a brief speculation, predicted oscilations in the levels of FFA could reflect the oscillations observed for GDF15, specially when early follow-up were included (Fig. 2 A).

Given its widespread expression across various tissues in the human body, further prospective clinical studies involving patients with obesity, carefully characterized by their pharmacotherapeutic profiles, circulating lipid profiles, body composition, skeletal muscle mass (and functionality), and liver (dys)function, are essential. Such studies will help clarify how baseline levels of GDF15 are influenced by the health status of these tissues and, in turn, how changes in GDF15 are associated with the clinical outcomes following surgical interventions for obesity, namely weight loss and reduction of MASLD.

## Conclusions

The role of GDF15 as a biomarker and potential modulator of metabolic processes in patients undergoing BMS remains an area of considerable complexity. While GDF15 levels are often elevated in states of obesity, its precise function and impact following BMS is not fully understood. This review underscores the inconsistency in findings across studies regarding the directionality of GDF15 changes post-surgery, namely in relation to liver dysfunction, a key concern in obesity-related metabolic diseases. The lack of comprehensive data on hepatic function in the cohorts examined limits a conclusive understanding of GDF15’s role in liver health post-BMS. The varying results across studies suggest that GDF15 may not be solely derived from adipose tissue but rather could be influenced by changes in other tissues, notably the liver and skeletal muscle, post-surgery. Prospective, well-characterized studies are necessary to unravel the complex interactions between GDF15 and the diverse tissue dynamics and crosstalk in obesity, ultimately guiding more personalized and effective treatments for patients with obesity and associated comorbidities.

## Data Availability

No datasets were generated or analysed during the current study.
